# Standardized patient methodology in mainland China: a nationwide survey

**DOI:** 10.1186/s12909-019-1630-y

**Published:** 2019-06-17

**Authors:** Hua-Xia Yang, Yuan Xu, Nai-Xin Liang, Wei Chen, Xue-Min Yan, Ping Yang, Zhi-Yuan Guan, Carol A. Pfeiffer, Qi Li, Jun Zhao, Hui Pan

**Affiliations:** 1 Department of Rheumatology and Clinical Immunology, Peking Union Medical College Hospital, Peking Union Medical College and Chinese Academy of Medical Sciences, Beijing, China; 2 Department of Thoracic Surgery, Peking Union Medical College Hospital, Peking Union Medical College and Chinese Academy of Medical Sciences, No.1 Shuaifuyuan, Wangfujing Street, Dongcheng District, Beijing, 100730 China; 3 Department of Cardiovascular Disease, Peking Union Medical College Hospital, Peking Union Medical College and Chinese Academy of Medical Sciences, Beijing, China; 4 Department of Gastroenterology, Peking Union Medical College Hospital, Peking Union Medical College and Chinese Academy of Medical Sciences, Beijing, China; 5 Department of Education, Peking Union Medical College Hospital, Peking Union Medical College and Chinese Academy of Medical Sciences, No.1 Shuaifuyuan, Wangfujing Street, Dongcheng district, Beijing, 100730 China; 6 UConn Health, Farmington, CT USA

**Keywords:** Standardized patient, China, Nationwide survey, Difficulty, Payment

## Abstract

**Background:**

To describe the current status of standardized patient (SP) practice in mainland China.

**Methods:**

We conducted a nationwide survey in 2016. One hundred and eighty-three SP educators (SPEs) responded to the questionnaire, representing 80 medical centers from 25 provinces in mainland China. All of these centers were affiliated with China Standardized Patients Practice Teaching Guidance. In the survey, we assessed the methods of SPs’ recruitment, hourly wage, how SPs were used and challenges of SP role. We also compared these data among the 4 different regions in China.

**Results:**

In mainland China, the most frequent range of SPs’ age was between 30 and 40 years (24.8%). The SPs were usually recruited by recommendations from the SPEs or a current SP (43.8%), as well as advertising in the hospitals (43.8%). The mean hourly wage was US$12.60 for teaching activities and US$18.82 for medical examinations. The median frequency for training SPs was 12.9 times per year. The SPs were used in areas such as internal medicine (89.6%), surgery (79.2%) and pediatrics (56.3%). The most challenging parts for the SPs were to remember all of the key points of the cases (51.9%) and portraying the emotions of the case (51.9%). Almost half of the SPs reported that, when interacting with medical students, they had difficulty in providing feedback in consistent with students’ learning objectives. SPs’ gender, age, rewards and scenarios playing were different significantly among the 4 geographic regions in China (*P* < 0.05).

**Conclusions:**

This survey provided the reliable data on the current situation of SP application in China. SP activities have had an encouraging progress but regional development imbalance.

## Background

A standardized patient(SP), also known as simulated patient, sample patient, or patient instructor, is an individual trained to act as a real patient in order to simulate a set of clinical symptoms or problems. Standardized patients have been widely used to support teaching and evaluation of medical students in developed countries since Barrows’ original description in 1968, and studies have reported the validity and reliability of the use of SPs [1–3].

Although SPs are employed extensively in the developed countries, little is known about how individual schools use and evaluate SPs in developing countries [4]. An initial survey conducted in 2009 described the functions and program structures of SP in US and Canada [5]. In 2010, a Japanese survey demonstrated that SP satisfaction is high but challenges in case mastery and feedback tasks are evident. [6]. An European study proposed a clear need of collaboration among different centers [7]. SPs were introduced in China for medical education in 1991 by Paula Stillman [8] and were officially put into use in medical teaching and assessment in 1994. Although a growing number of medical centers have launched SP programs since then, there is still a lack of descriptive data on the ways in which SPs are used in China.

In 2016 we conducted a national survey in China to understand how the SP programs are operated in individual schools in China. We collected information on the demographic features of the SPs, the methods of SP recruitment, the payment for SPs, and challenges of SP roles. We also described the differences of SP programs among the different geographic regions of China.

## Methods

### Survey design and data collection

We conducted the survey in mainland China in September 2016. Using the membership list of China Standardized Patients Practice Teaching Guidance (CSPC), we included 80 of the 86 medical centers, consisting medical colleges, academic hospitals and medical examination centers. The medical centers are located in 25 of the 34 administrative divisions of China (including provinces, municipalities, and autonomous regions). Questionnaires were sent to 243 standardized patient educators (SPEs), and 183 (75.3%) of them agreed to participate and completed the questionnaires. For the analysis, we combined “North” and “Northeast” regions into “North”, “Northwest” and “Southwest” regions into “West”, as these the respective regions have similar medical educational institutes. The final data comes from of 80 medical centers across all 4 geographic regions in China (North, East, South-central, and West). The study has been approved by the Ethics Committee of Peking Union Medical College Hospital (S-K705).

### Questionnaires

The survey questionnaire was drafted base on the published questionnaires for SP survey [9], and was modified according to the questionnaires for SP and SP educators developed in PUMCH. The questionnaire was then reviewed by experts, including three senior medical teachers, one epidemiologist and one statistician. A pilot survey of 10 SPEs was performed in PUMCH and another academic medical center. All scales showed adequate reliability with Cronbach’s Alpha (> 0.74).

### Statistical analysis

Continuous data were presented as mean (SD) or median (IQR) as appropriate, and categorical variables were presented as n (%). We compared between groups using one-way ANOVA or Kruskal-Wallis test for continuous variables and χ2 test for categorical variables. All *p* values were two-sided, and a *p* value of less than 0.05 was deemed significant. Analyses were performed with IBM SPSS Statistics (version 21.0, SPSS Inc., Chicago, IL, USA).

## Results

### Demographic features

Among the 80 medical centers affiliated with CSPC, 48 centers have provided SP-based medical education and examinations, indicating that more than half of the medical centers (60%) have employed the SP program. The median number of SPs in each center was 18 (14) persons. And the median launch time of SP programs in the medical centers was 5 (6) years. The ratio of female:male SPs was 2.1:1. The most frequent age range of the recruited SPs was between 30 and 40 years (24.8%), followed by 20–30 (18.4%), 50–60 (18.0%), 40–50 (17.8%), 16–20 (16.1%) and 60–80 years (4.8%) (Table 1).

**Table 1:** Characteristics of standardized patients according to geographic areas

	Total (*n* = 809)	North (*n* = 275)	East (*n* = 218)	South-central (*n* = 218)	West (*n* = 98)	*p* value
Age group						
16–20 years	130(16.1%)	64(23.3%)	53(24.3%)	13(6.0%)	0	**< 0.001**
20–30 years	149(18.4%)	18(6.5%)	30(13.8%)	55(25.2%)	46(46.9%)	**< 0.001**
30–40 years	201(24.8%)	77(28.0%)	51(23.4%)	58(26.6%)	15(15.3%)	0.077
40–50 years	144(17.8%)	62(22.5%)	27(12.4%)	39(17.9%)	16(16.3%)	0.033
50–60 years	146(18.0%)	41(14.9%)	43(19.7%)	47(21.6%)	15(15.3%)	0.207
60–80 years	39(4.8%)	13(4.7%)	14(6.4%)	6(2.8%)	6(6.1%)	0.306
Gender						
Male	263(32.5%)	82(29.8%)	60(27.5%)	75(34.4%)	46(46.9%)	**0.005**
Female	546(67.5%)	193(70.2%)	158(72.5%)	143(65.6%)	52(53.1%)	**0.005**

Among the 183 SPEs participated in this survey, 107 were female (58.5%) and 76 were male (41.5%), corresponding a female: male ratio of 1.4:1. The mean age of SPEs was 45.2(14.3) years. Our data showed that SPEs comprised a diverse group of professionals including doctors (109, 60.9%), medical administration staffs (47, 26.3%), nurses (6, 3.4%), medical students (2, 1.1%), and others (15, 8.4%).

### SPs’ recruitment and rewards

In mainland China, SPs were recruited in various ways. The most common way of recruiting SP members were referrals by the SPEs or a current SP (21, 43.8%). Other ways of recruitment included advertisement posting in the hospitals (21, 43.8%), recruitment among hospital patients (17, 35.4%) and through public media channels (16,33.8%). In 42 (87.5%) of the 48 medical centers, they used more than 3 methods to recruit SPs.

The rewards of SPs varied by the different medical activities performed. The mean hourly wage was RMB $85.7 (approximately US $12.60) for teaching activities, while a higher hourly wage of RMB $ 128.0 (approximately US $18.82) was paid for medical examinations (Table 2).

**Table 2:** SPs’ recruitments and rewards according to geographic areas

	Total (*n* = 48)	North (*n* = 19)	East (*n* = 10)	South-central (*n* = 13)	West (*n* = 6)	*p* value
Recruitment						
Propaganda among the patients	17(35.4%)	7(36.8%)	3(30.0%)	4(30.8%)	3(50.0%)	0.635
Recommend by SP educators	21(43.8%)	8(42.1%)	5(50.0%)	7(53.8%)	1(16.7%)	0.474
Propaganda through media channels	16(33.8%)	4(21.1%)	6(60.0%)	5(38.5%)	1(16.7%)	0.145
Post advertisements in the hospitals	21(43.8%)	6(31.6%)	4(40.0%)	7(53.8%)	4(66.7%)	0.389
Recommend by those who were already SPs	21(43.8%)	7(36.8%)	6(60.0%)	7(53.8%)	1(16.7%)	0.288
Rewards (RMB per hour)						
Teaching activities	85.7(41.5)	136.8(61.4)	58.3(34.2)	62.8(31.2)	37.5(17.0)	0.003
Medical examinations	128.0(62.3)	211.4(137.5)	70.8(42.8)	94.4(38.2)	60.0(28.3)	**0.011**

### SPs’ training and application

In the responding medical centers, the median frequency of training for SPs was 12.9 times per year. The most frequent way of SP training was by giving lectures, with a median frequency of 8.1 times per year, followed by clinical practices (3.1 times per year) and video training (1.8 times per year). The well-trained SPs were certified to participate in medical education. Most of them were used to train medical students in medical history taking and physical examination during the pre-clinical stage. The SPs were used in areas including internal medicine (89.6%), surgery (79.2%) and pediatrics (56.3%). The SPs were employed in the initial visit scenario in over 90% of the centers. Meanwhile, other different scenarios, including return visit (33, 68.8%), patient education (21, 41.7%), telling bad news (14, 29.2%) and conversation with relatives (20, 41.7%) were also designed for advanced medical education (Table 3). Although 40 (83.3%) centers agreed that it is very important or important to apply SPs for physical examination, only a few centers (9, 18.8%) used that. Further, only 9 (18.8%) centers considered it was feasible to apply female SPs for breast examination. The female: male SP ratio for rectal examination was also low, at a rate of 4.2% (*n* = 2) female versus 16.7% (*n* = 9) male. In this survey, 28 (58.3%) medical centers reported their current use of SPs in OSCE, and the other 20 centers were in preparation to apply SPs in OSCE.

**Table 3:** SPs’ training and application according to geographic areas

	Total (*n* = 48)	North (*n* = 19)	East (*n* = 10)	South- central (*n* = 13)	West (*n* = 6)	*p* value
Training frequencies (time per year)	12.9(10.5)	11.7(10.8)	15.7(14.9)	9.4(6.6)	17.6(16.2)	0.594
Lectures	8.1(7.6)	5.0(4.2)	9.7(8.4)	7.6(6.3)	9.8(4.3)	0.216
Video training	1.8(2.0)	2.7(1.9)	3.0(3.6)	0.6(0.9)	0(0)	0.125
Clinical practice	3.1(2.5)	4.1(3.2)	3.0(2.7)	1.5(1.4)	4.4(2.7)	0.516
Application						
Clinical scenarios						
Internal medicine	43(89.6%)	17(89.5%)	8(80.0%)	12(92.3%)	6(100%)	0.618
Surgery	38(79.2%)	15(78.9%)	7(70.0%)	11(84.6%)	5(83.3%)	0.630
Pediatrics	27(56.3%)	10(52.6%)	6(60.0%)	8(61.5%)	3(50.0%)	0.940
Psychiatry	12(25.0%)	5(26.3%)	3(30.0%)	2(15.4%)	2(33.3%)	0.798
Gynaecology and obstetrics	24(50.0%)	8(42.1%)	6 (60.0%)	7(53.8%)	3(50.0%)	0.813
Neurology	11(22.9%)	6(31.6%)	1(10.0%)	2(15.4%)	2(33.3%)	0.469
Emergency	19(39.6%)	10(52.6%)	3(30.0%)	5(38.5%)	1(16.7%)	0.382
Scenario topics						
First visit	44(91.7%)	17(89.5%)	8(80.0%)	13(100%)	6(100%)	0.304
Return visit	33(68.8%)	12(63.2%)	6(60.0%)	7(53.8%)	5(83.3%)	0.669
Patient education	20(41.7%)	9(47.4%)	3(30.0%)	4(30.8%)	3(50.0%)	0.843
Telling bad news	14(29.2%)	7(36.8%)	3(30.0%)	4(30.8%)	0(0.0%)	0.387
Conversation with relatives	20(41.7%)	11(57.9%)	6(60.0%)	2(15.4%)	1(16.7%)	**0.034**

### Training challenges in SPs’ performance and feedback

SPEs identified several challenges in SPs’ performance, including to have the SPs to remember all the key points of the case (95, 51.9%) and to portray the emotions of the patient (95, 51.9%). followed by role shaping (76, 41.5%) and the use of appropriate vocabularies (23, 12.6%). Training SPs to give appropriate feedback to medical students is an advanced stage of SP training and is a challenge for SPEs. Indeed, SPEs reported that almost half of the SPs had difficulty in providing feedback in consistent with the student’s learning goals. Other challenges expressed by SPEs included consistently maintaining the emotion of the case throughout the role playing (76, 41.5%), avoiding general comments (69, 37.7%), expressing well-balanced positive and negative points (48, 26.2%), and emotional control (29, 15.8%) (Table 4).

**Table 4:** Training challenges expressed by SPEs according to geographic areas

	Total (*n* = 183)	North (*n* = 81)	East (*n* = 37)	South- central (*n* = 40)	West (*n* = 25)	*p* value
Training challenges in SP’s performance						
Role shaping	76(41.5%)	34(42.0%)	16(43.2%)	16(40.0%)	10(40.0%)	0.990
Remember all the key points of the cases	95(51.9%)	46(56.8%)	14(37.8%)	19(47.5%)	9(36.0%)	0.140
Using proper words	23(12.6%)	12(14.8%)	5(13.5%)	4(10.0%)	2(8.0%)	0.773
Portray the emotions of the cases	95(51.9%)	39(48.1%)	21(56.8%)	21(52.5%)	14(56.0%)	0.806
Training challenges in SPs’ feedback						
Remember the feeling while acting	76(41.5%)	41(50.6%)	10(27.0%)	17(42.5%)	8(32.0%)	0.075
Avoid the general comments	69(37.7%)	32(39.5%)	11(29.7%)	17(42.5%)	9(36.0%)	0.674
Express well-balanced positive and negative points	48(26.2%)	21(25.9%)	7(18.9%)	12(30.0%)	8(32.0%)	0.626
Providing feedback in consistent with the study objects	93(51.1%)	44(54.3%)	16(44.4%)	22(55.0%)	11(44.0%)	0.632
Emotional control	29(15.8%)	17(21.0%)	5(13.5%)	2(5.0%)	6(24.0%)	0.097

### Comparisons among geographic regions

Data were compared among the different geographical regions in China (shown in Tables 1-4). We found SPs’ gender, age, rewards and SPs’ application scenarios were different significantly among the 4 geographic regions (*P* < 0.05). More female SPs were recruited in the North/East regions than the South-central/West regions. The highest rewards of SPs (teaching activities and medical examinations) were reported in the North region. As for scenario topics for SP cases, the North/East regions designed more cases in regards to the conversations with relatives. There were no significant differences in the rest of the items collected in this study (*P* > 0.05).

## Discussion

Limited studies are available to describe the development of SP programs and variation in working conditions, especially in Asian countries. Our survey provided the reliable data of current SP practices applied in the medical teaching and assessment in China. Eighty out of 86 medical centers affiliated with CSPC participated in the survey, indicating a very high response rate. We analyzed demographic features and several operational aspects of the SP practices including recruitment, rewards, application and training challenges. To our knowledge, the present study represented the first survey to provide data on the current situation of SP application in China. The survey indicated an unbalanced development of the status of SP industry among different regions in China. SP educators should strengthen cooperation among colleagues in China, as well as those outside China.

Different Chinese medical centers had similar ways of recruiting SPs, with recommendation by the practitioners being the most common. Recruitment using media channel was effective but not widely used in China. The use of SPs varied largely across centers, covering areas such as internal medicine, surgery, pediatrics, psychiatry, gynecology and obstetrics, neurology and emergency medicine. However, some traditional scenarios (such as first and return visits) and patient interactions such as patient education, telling bad news and conversation with relatives were also included for SP performance in most centers. It is noted that there were much more centers in the northern and eastern areas utilized SPs in scenarios practice for interactions with relatives. This may be due to the more developed SP programs in these regions that covered a wider range to topics.

One of the unique finding in this survey was the payment of SPs. Compared to developed countries, the employment of SP practice in mainland China is still in early development. SPs in Canada and the USA were paid at $16/h [5], while in China it varied from $6 to $35/h, suggesting a lack of mature and consistent reward system. Interestingly, SPs were tended to be paid higher in the medical centers that have the SP program launched earlier, suggesting medical centers with mature SP programs are willing to pay higher to retain the SPs. In regards to the regional difference, Northern centers tended to pay higher to SPs than the rest of the regions.

Chinese SPEs expressed that having the SPs to remember the key points and portraying emotion that matched the case were the challenging. Training SPs to give appropriate feedback to medical students was also a challenge, as 51.1% SPEs expressed that having the SP to provide feedback in consistent with medical student’s learning objective was the most difficult part of the job. Several scales such as Maastricht Assessment of Simulated Patients (MASP) [10] have suggested helpful ways to assess SP performance. In our survey, only a few centers in China reported the application of such evaluation tools, reflecting the need to improve the assessment system of SP training in China.

Objective structural clinical examination (OSCE) was proposed in 1975 and now is a well-recognized approach to evaluate clinical skill performance and competence in skills such as clinical examination, communication etc. OSCE was introduced to China in 1990s, and there were 40 medical centers using OSCE in medical examination by 2013. It should be noted that most of the Chinese medical centers tended to use SP cases for medical history taking. The application of SPs in physical examination was not widely accepted. Only a few centers are using SP in such area, although some of the centers expressed that they were in establishment to use SP in physical examination. This situation is different from Europe, in which the application rate of SP in physical examination was as high as 73.8% [7]. Traditional Chinese culture might be the main reason for the less use of SP in physical examination, in which physical examination is considered highly private, especially for females.

Our study revealed potential for cooperation among Chinese medical centers, as only 14 (29.2%) had the experiences of sharing SP education resources. A majority of the centers, 29 (60.4%), designed cases independently. The independent operation of the SP programs could potentially increase the overall costs. In 2016, CSPC was established in mainland China. In the future, it is believed that more collaboration and exchange activities will be conducted among Chinese medical centers, as well as with institutions outside China.

Although this study represents the first national-wide survey conducted in China, it has its limitations, such as the selection bias. This study was based on the questionnaires received from the SPEs, the results may not be reflecting the complete situation of SPs. Additionally, some medical centers in undeveloped districts were not participated in this survey, which could overestimate the development status of the SP program in China. Future studies are needed to address these issues in order to provide a more complete data set of SP practice in China.

## Conclusions

SP activities in China have had an encouraging progress, although there are still some aspects that remain to be improved. More educational resources should be provided to support the development of SP programs in China.

## Data Availability

The datasets generated and analyzed during the current study are available from the corresponding author on reasonable request.

## Abbreviations

MASP: Maastricht Assessment of Simulated Patients
SP: Standardized patient
